# Cysteamine Eye Drops in Hyaluronic Acid Packaged in Innovative Single-Dose Systems: Stability and Ocular Biopermanence

**DOI:** 10.3390/pharmaceutics14102194

**Published:** 2022-10-15

**Authors:** Ana Castro-Balado, Enrique Bandín-Vilar, Andrea Cuartero-Martínez, Laura García-Quintanilla, Gonzalo Hermelo-Vidal, Xurxo García-Otero, Lorena Rodríguez-Martínez, Jesús Mateos, Manuela Hernández-Blanco, Pablo Aguiar, Irene Zarra-Ferro, Miguel González-Barcia, Cristina Mondelo-García, Francisco J. Otero-Espinar, Anxo Fernández-Ferreiro

**Affiliations:** 1 Pharmacy Department, University Clinical Hospital of Santiago de Compostela (SERGAS), 15706 Santiago de Compostela, Spain; 2 Clinical Pharmacology Group, Health Research Institute of Santiago de Compostela (IDIS), 15706 Santiago de Compostela, Spain; 3 Pharmacology, Pharmacy and Pharmaceutical Technology Department, Faculty of Pharmacy, University of Santiago de Compostela (USC), 15782 Santiago de Compostela, Spain; 4 Molecular Imaging Group, Health Research Institute of Santiago de Compostela (IDIS), 15706 Santiago de Compostela, Spain; 5 Microbiology Department, University Clinical Hospital of Santiago de Compostela (SERGAS), 15706 Santiago de Compostela, Spain

**Keywords:** cystinosis, ophthalmic administration, cysteamine, compounded formulation, PET

## Abstract

Cystinosis is a rare genetic disorder characterized by the accumulation of cystine crystals in different tissues and organs causing, among other symptoms, severe ocular manifestations. Cysteamine eye drops are prepared in hospital pharmacy departments to facilitate access to treatment, for which vehicles that provide adequate biopermanence, as well as adaptable containers that maintain its stability, are required. Difficulties related to cysteamine preparation, as well as its tendency to oxidize to cystamine, show the importance of conducting rigorous galenic characterization studies. This work aims to develop and characterize an ophthalmic compounded formulation of cysteamine prepared with hyaluronic acid and packaged in innovative single-dose systems. For this task, the effect of different storage temperatures and the presence/absence of nitrogen on the physicochemical stability of the formulation and its packaging was studied in a scaled manner, until reaching the optimal storage conditions. The results showed that 0.55% cysteamine, prepared with hyaluronic acid and packaged in single-dose containers, is stable for 30 days when stored at −20 °C. In addition, opening vials every 4 h at room temperature after 30 days of freezing maintains the stability of the cysteamine formulation for up to 16 h. Moreover, ocular biopermanence studies were conducted using molecular imaging, concluding that the biopermanence offered by the vehicle is not affected by the freezing process, where a half-life of 31.11 min for a hyaluronic acid formulation stored for 30 days at −20 °C was obtained, compared with 14.63 min for 0.9% sodium chloride eye drops.

## 1. Introduction

Cystinosis is considered a rare autosomal recessive lysosomal disease that affects approximately 100,000–200,000 people in the general population [1,2]. It is characterized by the lysosomal accumulation of cystine crystals, the disulfide of the amino acid cysteine, with low solubility in water. Mutations in the CTNS gene, which encodes cystinosin, the carrier that transports cystine from the lysosome, are responsible for this cumulative process [3]. The presence of cystine crystals in different tissues leads to the progressive damage and dysfunction of multiple organs, such as the pancreas, brain, thyroid, kidneys, and eyes [4]. The most frequently described ocular manifestation is the deposition of cystine crystals in the cornea, but it also affects other eye structures, causing visual impairment and eventually, blindness [5,6,7]. 

Currently, the aminothiol cysteamine remains the only available treatment of cystinosis [8], although other pathways have been recently explored to expand therapeutic options [9,10,11,12]. Cysteamine lowers intracellular levels of cystine by forming a cysteamine–cysteine mixed disulfide, which resembles lysine and leaves the lysosome using a “lysine transport system” [8,13]. The authorization of oral cysteamine (Cystagon^®^) in 2012 by the Food and Drug Administration (FDA), followed by the delayed-release formulation years later (Procysbi^®^), has completely changed the management and prognosis of cystinosis [14,15,16]. However, systemic cysteamine has a low ocular bioavailability due to the lack of corneal vascularization, so it must be administered by the ophthalmic route. Cystaran^®^ 0.44% eye drops received FDA approval in 2012 as an orphan drug, requiring administration of one drop per eye every waking hour [17], which complicates patient compliance. To improve this aspect, Cystadrops^®^ 0.55% was subsequently approved by the European Medicines Agency (EMA) and the FDA in 2017 and 2021, respectively [18,19]. This formulation contains sodium carboxymethylcellulose that provides high viscosity, thus achieving a longer residence time on the ocular surface and allowing its administration four times a day [19].

Commercial cysteamine preparations are not available in most countries, as access is sometimes delayed due to mandatory procedures and authorizations. In this scenario, hospital pharmacy departments (HPD) are responsible for preparing cysteamine ophthalmic compounded formulations as a therapeutic alternative [20]. These formulations need to be properly developed with appropriate galenic characterization studies prior to their translation into clinical practice [21]. Cysteamine is an easily oxidizable molecule whose exposure to the air reduces its shelf life, since the thiol functional group immediately reacts with oxygen to form a disulfide called cystamine, which is ineffective in the treatment of cystinosis [21,22,23]. This process occurs quickly in air and solution [24], making it difficult to develop adequate determination methods that allow the simultaneous detection of cysteamine and cystamine [25]. Strategies such as packaging under a nitrogen atmosphere, adding antioxidants, reducing the pH, and lowering the temperature in storage, effectively increase its stability [26,27,28]. 

Classical compounded formulations of cysteamine eye drops are produced in HPD with 0.9% sodium chloride (NaCl), but these require frequent instillations while the patient is awake due to its low ocular biopermanence. To optimize the formulation and avoid these difficult dosage schedules, our group developed and characterized a bioadhesive cysteamine hydrogel with high ocular permanence, using hyaluronic acid 0.4% as a vehicle [6,20,29]. However, after four years of producing this formulation in our center, we have detected in the internal quality controls that important parameters, such as the molecular weight of the sodium hyaluronate used (supplied by the same distributor authorized by the Spanish Agency of Medicines and Medical Devices), exhibited important variations of up to 40% in the different batches produced. In addition, other substances, including proteins, nucleic acids, metals, and chlorate content, have been detected [30,31]. According to previous publications, this could significantly interfere with compounding and stability studies using different batches, as they would no longer be reproducible [32,33,34,35]. To overcome this problem, we have decided to use commercialized eye drops that contain 0.4% hyaluronic acid (Aquoral^®^). This choice offers the advantages of a constant source, a dependable purification technique, and a certified molecular weight in the hyaluronate used. 

When transferring compounded formulations to clinical practice, it is important to take into account the comfort of the patient and the pharmacy staff in charge of the preparation. Therefore, packaging in single-dose systems would allow the use of one disposable container per day, while the rest of the dispensed batch could be kept at low-temperature conditions that favor the stability of the active ingredient, in this case, cysteamine. The use of one single-dose container per day would facilitate its handling and transportation by patients, also reducing the number of visits to the hospital for dispensing treatment. When using these single-dose containers, it is necessary to devise a system that allows easy and fast filling, but without entailing a workload in the formulation production circuit. At the same time, it is important to consider whether storage at low temperatures can negatively affect the physical properties of the compounded formulation, or the properties of the container.

The objective of this work is to develop and characterize an ophthalmic compounded formulation of 0.55% cysteamine, packaged in single-dose containers, for its elaboration from HPD. The stability study of the formulation and its final container was carried out in a scaled manner under different storage conditions (with/without nitrogen saturation, refrigerated or frozen) for 30 days to achieve the most optimal form of conservation. Their chemical (cysteamine and cystamine content, pH), physical (osmolality and viscosity), and microbiological stability, as well as packaging elasticity after storage at low temperatures, were evaluated. An in-use stability study was also conducted at room temperature to simulate conditions for opening the eye drops prior to administration. Finally, a preclinical ocular biopermanence study was carried out by PET/CT imaging.

## 2. Materials and Methods

### 2.1. Materials 

Cysteamine hydrochloride (cysteamine purity 97%) was obtained from Apollo Scientific (Stockport, UK) and 1-Heptanesulphone acid sodium salt was obtained from Sigma Aldrich (St. Louis, MO, USA). Cystamine dihydrochloride (Cystamine purity 97%) was obtained from Thermo Fisher Scientific (Geel, Belgium). Aquoral^®^ eye drops (0.4% *w*/*v* % hyaluronic acid (HA), sodium chloride, sodium citrate, citric acid monohydrate, water for injectable preparations) were purchased from Esteve (Barcelona, Spain). Acetonitrile UHPLC-MS grade and acetic acid were purchased from VWR Chemicals (Radnor, PA, USA). Balanced Salt Solution (BSS^®^) was purchased from Alcon (Geneva, Switzerland). Ultrapure water from MilliQ, Merck Millipore (Madrid, Spain) was used. As a packaging material, COL Eye Drops System^®^ made with polyvinyl chloride (PVC) and di-n-octyl phthalate/di(2-ethylhexyl) phthalate (DOP/DEHP), free from Biomed Device (Modena, Italy), were used as single-dose containers.

### 2.2. Elaboration and Packaging of Cysteamine Hydrochloride Sterile Solutions

Initially, 275 mg of cysteamine were dissolved in 10 mL of BSS via magnetic stirring. To achieve a cysteamine concentration of 0.55%, sufficient Aquoral^®^ (AQ) was used as a diluent. Finally, sterilizing filtration was performed with a 0.22 μm membrane filter (Stericup^®^ Merck Millipore ExpressTM PLUS) under vacuum. The compounded formulation was produced in triplicate.

Single-dose systems were filled under aseptic conditions in a horizontal laminar flow cabinet by generating an initial vacuum using the coupled syringe, connections, and filters, according to the manufacturer’s instructions [36,37]. Successive aspirations and expulsions of the air contained inside the system were carried out until the complete collapse of the single-dose containers was achieved. Through the lateral connection, 30 mL of each of the formulations were aspirated with the syringe incorporated in the system. The previously generated vacuum enables a homogeneous filling of the single-dose units (approximately 1.3–1.5 mL per vial) once the corresponding stopcocks are opened. In the case of single-dose vials saturated with nitrogen, an epidural needle was immersed in the 30 mL formulation contained in the syringe after the plunger was withdrawn. Nitrogen sparging was performed through the needle connected to a 0.22 μm filter for a total of 5 min per system, plugging the syringe opening with the plunger as much as possible. The filling of the vials was carried out as mentioned above.

Finally, each single-dose container was individually sealed with a heat-sealing gun provided by the manufacturer, and its correct closure was individually checked.

### 2.3. Cysteamine and Cystamine Quantification

The quantification of cysteamine and cystamine in the compounded formulations was analyzed by ultra-high-performance liquid chromatography (UHPLC) using a modified version of the method proposed by Kim et al. [38]. The analysis was performed with a UHPLC coupled with photodiode array detection (PDA), using an ACQUITY UPLC H-Class System (Waters^®^) with ACQUITY PDA detector (Waters^®^).

We employed an ACQUITY^®^ BEH C18 column (2.1 × 50 mm, 1.7 µm, Waters^®^) at a temperature of 45 °C. The mobile phase was a mixture of aqueous 4 mM sodium 1-heptane sulfonate:acetonitrile in gradient mode, as shown in Table 1. The wavelengths used for the detection of cysteamine and cystamine were 215 nm and 244 nm, respectively. Data were collected and processed with Empower 3 Software (2002−2019 Waters^®^) Application Manager.

All test samples were diluted 1:50 with 0.1% acetic acid and analyzed by UHPLC, setting the injection volume to 5 µL.

### 2.4. Storage Stability Study

Single-dose vials, with and without nitrogen saturation, were stored, protected from light, in a refrigerator (2–8 °C) or freezer (−20 °C). Initially, the storage stability study was carried out for a period of 30 days. Storage stability tests were performed in triplicate and conducted on days 0, 3, 7, 15, 21, and 30. All samples were kept at room temperature for at least 30 min prior to analysis to avoid measurement errors due to temperature variations.

Based on the most stable storage conditions, an in-use stability study was carried out once the vials were opened.

#### 2.4.1. Determination of pH and Osmolarity

The pH of the formulations was determined with a BasiC20^®^ pH meter, while the osmolarity was measured with a cryoscopic freezing point osmometer (OsmoSpe-cial1^®^) in 150 μL aliquots. Each determination was carried out in triplicate on days 0, 3, 7, 15, 21, and 30.

#### 2.4.2. Viscosity Tests

The viscosity of cysteamine compounded formulations was determined on days 0, 15, and 30 in triplicate, with a rotational viscometer (Anton Paar ViscoQC 300^®^ with PTD 80 Peltier Temperature). For this, 2 mL of each formulation were introduced into the equipment, and the measurement was carried out at 25 °C and 100 revolutions per minute (rpm). A one-way ANOVA was carried out to determine if there were significant differences.

In addition, a viscosity speed-scan test was also performed at different rpm in order to analyze the rheological behavior of cysteamine eye drops in hyaluronic acid. 

#### 2.4.3. Microbiological Stability

Each formulation was analyzed on days 0 and 30 to determine microbiological stability. Aliquots of 1.5 mL were added to plates containing blood agar, Sabouraud agar, and fluid thioglycolate medium. Subsequently, plates were cultured at 37 °C for 48 h, 15 days, and 10 days, respectively. At the end of each incubation period, the plates were inspected for any signs of microbiological growth.

#### 2.4.4. Statistical Analysis

The Pharmaceutical Codex was used to establish the expiry date of the compounded formulations, which was set at a reduction ≥10% of cysteamine with respect to the initial concentration [39]. Changes in pH and osmolality were considered unacceptable if their values exceeded the acceptance criteria for ophthalmic applications [40,41]. Microbiological stability was considered acceptable when no microbial growth occurred in the cultured samples. 

The results of the different assays were compared by multivariate analysis of variance using Graph Pad Prism^®^ v.9.0.1 software.

#### 2.4.5. Elasticity of Single-Dose Containers

The effect of refrigerated and frozen storage on the mechanical strength of single-dose containers was studied in triplicate, measuring its elastic properties using a precision universal testing machine, Shimadzu Autograph AGS-X Series, with a load cell of 1 kN using Trapezium X material testing operation software (Shimadzu corporation, Kyoto, Japan). Measurements were made at time 0 and after 30 days of storage in the refrigerator or freezer. After emptying the content, the head of the single dose was cut and positioned with the help of adjustable clamps in the press at each of its ends. The tensile test speed was 0.2 cm/s, and force-displacement curves were recorded during the experiment to determine elastic modulus (Young’s modulus). A one-way ANOVA was carried out to determine whether there were significant differences.

### 2.5. In-Use Stability Study

Once the storage stability data was known, the in-use assay was carried out using vials previously stored under the best conditions. These vials were opened at room temperature, protected from light, every 4 h for 24 h, removing two drops per opening, except for during 8 h of sleep. The concentration of cysteamine and cystamine was measured at each of the openings.

### 2.6. In Vivo Evaluation of the Residence Time on the Ocular Surface

In vivo studies were carried after storage stability data were available, using vials previously stored under optimal conditions. The aim of this assay was to determine whether the freezing/thawing process significantly affects the ocular biopermanence properties of the formulation. An initial measurement was made on the day of preparation, followed by a measurement at day 30 of vials stored in the freezer. In addition, NaCl 0.9%, without bioadhesive properties, was used as a control formulation.

These studies were performed on four male Sprague Dawley rats (8 eyes), with an average weight of 250 g, supplied by the University of Santiago de Compostela (Santiago, Spain). The animals were kept in individual cages, with free access to food and water, in a room under controlled temperature (22 ± 1 °C) and humidity (60 ± 5%) and with day-night cycles regulated by artificial light (12/12 h). The animals were treated according to the guidelines for laboratory animals (National Research Council (US) Committee for the Update of the Guide for the Care and Use of Laboratory Animals, 2011; The Association for Research in Vision and Ophthalmology, 2014). Experiments were approved by the Animal Experimentation Ethics Committee of the Health Research Institute of Santiago de Compostela (2021/430700).

To quantitatively evaluate ocular permanence, radiolabeling of the formulation was performed following the method described in previous works of our group [6,42]. Positron emission tomography (PET) images were acquired using the ALBIRA PET/CT preclinical imaging system (Bruker Biospin, Woodbridge, CT, USA). Prior to image acquisition, the animals were anesthetized in a gas chamber with an administration of 3% isoflurane in oxygen, an anesthetic method that continued during acquisition through an inhalation mask. Throughout the procedure, the respiratory rate was monitored, and after each examination, the animals were awakened.

Once asleep, 7.5 µL of each formulation previously labeled with ^18^F-fluorodeoxyglucose (^18^F-FDG) (formulation:FDG ratio 10:1) was instilled with an automatic micropipette into the conjunctival fornix. The range of radioactivity administered in each eye was between 0.15–0.25 MBq. Immediately after instillation, a 10 min individual PET was acquired, and at 30, 60, 90, and 120 min. Once the images were reconstructed using 6 iterations, they were analyzed with Amide’s Medical Image Data Analysis Tool [43], manually drawing regions of interest (ROI) with a spherical shape of 15 × 15 × 15 mm delimiting the signal area in each eye. Finally, the percentage of activity (%) at a certain time was corrected for radioactive decay with the help of a one compartment model using GraphPad Prism^®^ 8 v.8.2.1 software. A non-compartmental analysis was also performed by calculating the remaining percentage of the formulations versus time.

## 3. Results

### 3.1. Cysteamine and Cystamine Quantification

**Figure 1:**
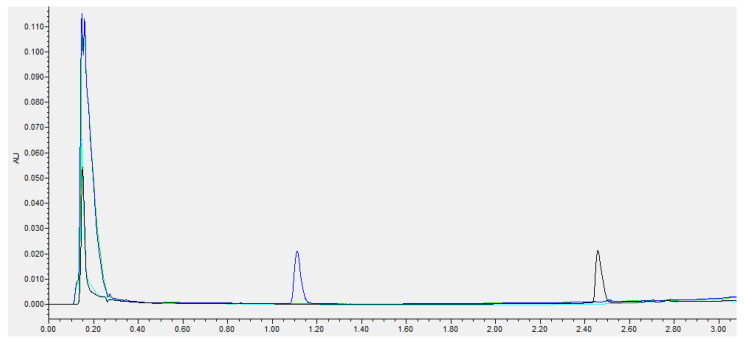
UHPLC chromatogram overlays showing the elution profiles of cysteamine at 215 nm (dark blue) and its oxidation product cystamine at 244 nm (black) in the studied matrix, Aquoral^®^ (green at 215 nm, light blue at 244 nm). Data were collected and processed with Empower^®^ 3 Software Application Manager.

The UHPLC-PDA determination method employed is highly specific and allows for the simultaneous detection of both cysteamine and cystamine, in only 6 min. A narrow, symmetrical, and well-defined chromatographic peak of cysteamine was obtained with an elution time of 1.12 min, while for cystamine, it was obtained at minute 2.46 (Figure 1). The analytical method was validated for linearity, accuracy, precision, and detection and quantification limits for cysteamine and cystamine. A linear calibration curve was obtained over a cysteamine and cystamine concentration range of 0.5–10 mg/mL (R^2^ = 0.999). The limit of detection (LOD) and limit of quantitation (LOQ) were 0.25 mg/L and 0.5 mg/L, respectively, for both compounds. Compliance with the analytical validation standards of the European Medicines Agency (EMA) was also ascertained [44].

### 3.2. Storage Stability Study

**Figure 2:**
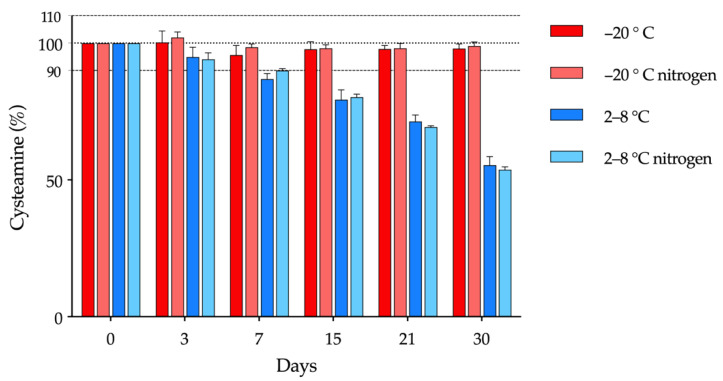
Cysteamine content with respect to the initial concentration throughout the study for each of the temperature settings (refrigeration/freezing) and in the presence/absence of nitrogen.

The variation in the cysteamine concentration of the compounded formulations made with AQ and packaged in single-dose containers over time under the different experimental conditions is shown in Figure 2. Cysteamine concentrations fell below 90% after day 7 when kept refrigerated, without noting any relevant differences in terms of saturation with nitrogen (n.s., two-way ANOVA followed by Tukey´s multiple comparison test). On the other hand, stability increased considerably when single-dose containers were stored in the freezer, having a physicochemical stability of up to 30 days prior to opening. As observed for storage in the refrigerator, saturation with nitrogen did not affect the concentration of cysteamine. In parallel to the decrease in cysteamine concentration, there was an increase in cystamine concentration throughout the study, as shown in Figure S2 of the Supplementary Material.

Cysteamine showed zero-order degradation kinetics, since the rate of reaction was independent of drug concentration. The degradation rate constants (K) of formulations preserved at 2–8 °C, in which significant degradation occurred, were calculated using the Arrhenius Equation (1):

(1) $$K=A\cdot e^{-Ea/R\cdot T}$$

where *K* is the degradation rate constant; *A* is the frequency factor assumed independent of temperature, which represents the frequency of collisions between reactant molecules at a standard concentration; *Ea* is the activation energy (J/mol); *R* is the gas constant (8.314 J/mol·K); and *T* is the temperature expressed in Kelvin. Results are shown in Table 2.

#### 3.2.1. pH and Osmolality

**Figure 3:**
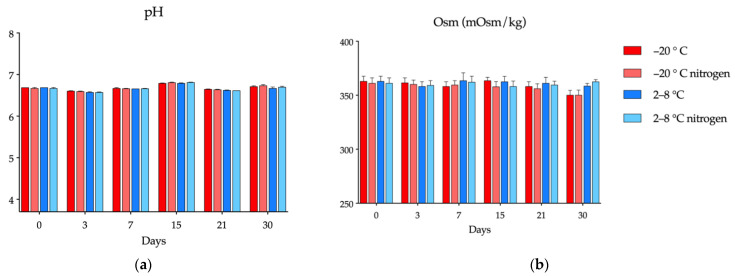
Variation in pH and osmolality (mOsm/kg) of the 5.5 mg/mL cysteamine formulation in Aquoral^®^ on days 0, 3, 7, 15, 21, and 30: (**a**) pH; (**b**) osmolality.

Variations in the pH of the cysteamine compounded formulations under the two preservation conditions over time are depicted in Figure 3a. With the use of the AQ as a vehicle, the pH remained stable (6.5–6.8) throughout the stability study under freezing or refrigeration. Likewise, nitrogen saturation did not cause variations with respect to the vials that contained oxygen. These constant pH values are probably due to some buffering effect exerted by the excipients (sodium citrate and citric acid monohydrate) present in the AQ eye drops [45]. 

Osmolality values (around 350 mOsm/Kg) of the formulations under the four experimental conditions remained between ±5% of the initial values over the study period (Figure 3b), being the dispersion of the values greater at day 30.

#### 3.2.2. Viscosity tests

**Figure 4:**
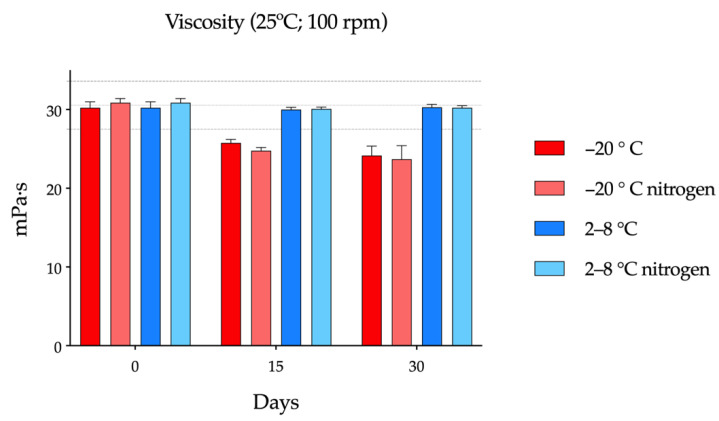
Viscosity variation measured at 25 °C and 100 revolutions per minute throughout the study period of the cysteamine formulations in Aquoral^®^ stored in a freezer (−20 °C)/refrigerator (2–8 °C) and with/without nitrogen saturation.

The initial viscosity of the formulations, measured at 25 °C and 100 rpm, was found to be around 30 mPa·s, as seen in Figure 4. On day 15 of the study, single-dose vials stored in the freezer showed a statistically significant drop in viscosity (*p =* 0.0098) with respect to that measured at day 0. On day 30, no statistically significant changes were observed with respect to the previous measurement on day 15, although the differences with respect to day 0 were maintained (*p =* 0.0057). On the other hand, for samples preserved in the refrigerator, viscosity hardly varied between days 0, 15, and 30. It was also observed that saturation with nitrogen did not introduce variations in the viscosity of the formulation when comparing vials kept at the same temperature.

The pseudoplastic behavior of HA has been observed regarding the present results, since the viscosity values decrease as the shear force increases. The viscosity values with respect to different rpm in all the formulations are represented in Figures S3–S5.

#### 3.2.3. Microbiological Stability

No microbial growth was observed in any of the compounded formulations during the storage period, which indicates that adequate storage of the samples was maintained under all study conditions.

#### 3.2.4. Elasticity of Single-Dose Containers

**Figure 5:**
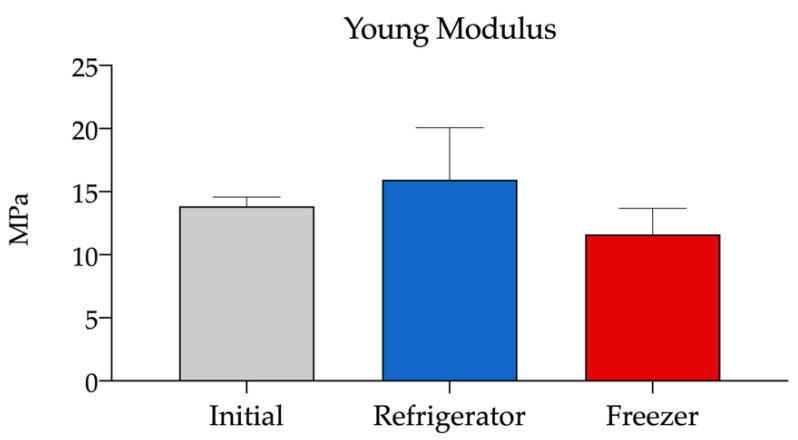
Young’s modulus of the COL Eye Drops^®^ vials used for the stability study at the initial time and after 30 days stored in the refrigerator (2–8 °C) and freezer (−20 °C).

Young’s modulus (*E*) is the ratio between the tensile stress (force per unit area) and the deformation of the solid material in the linear elastic region of the force-displacement curve. It is calculated using the following Equation (2):

(2) $$E=\frac{\sigma }{\varepsilon }$$

where *E* is the Young’s modulus, *σ* is the force per unit area, and *ε* is the proportional deformation. Young’s modulus values were obtained in this study by Trapezium X software directly from the slope of the tensile stress-deformation curve in the initial linear elastic region of the trace, considering the dimension of the probe used in the experiment. The results showed no statistically significant differences between the two storage temperatures of the containers over time (Figure 5). In addition, the change in volume of the content with freezing did not cause modifications in its elastic properties.

Considering these results, freezing without nitrogen is postulated as the best and most practical storage condition. With vials stored under these conditions for 30 days, the in-use stability study was evaluated, with the aim of determining their chemical stability after being opened, simulating their real-life use.

### 3.3. In-Use Stability Study

As in the storage assay, while the concentration of cysteamine decreased, cystamine concentration increased throughout the study. At the time of opening, a slight decrease in the cysteamine concentration was already observed, as well as a slight increase in the cystamine concentration, which is consistent with the degradation detected after 30 days of storage at −20 °C (Figure 2). After opening each single-dose container 5 times every 4 h at room temperature (16 h after removal from the freezer and initial opening), the concentration of cysteamine remained above 90% with respect to the initial concentration on day on which the formulation was prepared. These results indicate that single-dose vials stored for up to 30 days in the freezer can be used throughout a day, excluding the 8 h of sleep, once thawed and opened.

### 3.4. In Vivo Evaluation of the Residence Time on the Ocular Surface

**Figure 6:**
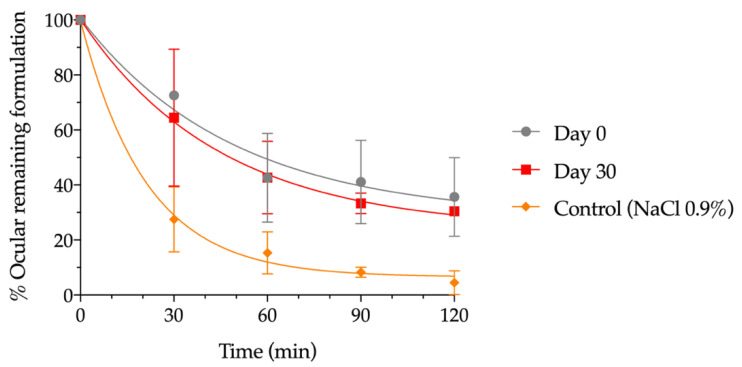
Percentage of formulation remaining on the ocular surface with respect to time zero determined by PET at day 0 and day 30 after storage in the freezer, as compared with NaCl 0.9%.

The residence time on the ocular surface of the pharmaceutical compounding was calculated immediately after its preparation and after being stored in the freezer for 30 days using small-animal PET imaging in rats. Figure 6 shows the permanence of these formulations from the eye compared to NaCl 0.9% (control). A strong signal immediately after instillation was observed and, after 120 min of contact, a considerable percentage of hydrogel remained on the ocular surface. This behavior is observed in a similar way, with no statistically significant differences, throughout the different PET images, for day 0 after formulation and day 30 after formulation, when stored at −20 °C. 

Data were fitted to a mono exponential equation dependent on time, as shown in Figure 6, and the pharmacokinetic parameters obtained by fitting to the one-compartmental model. A half-life of 33.86 min for day 0 after formulation and 31.11 min for day 30 after formulation, when stored at −20 °C, was obtained, demonstrating no influence of freezing on this parameter and showing an ocular residence time more than two-fold higher than for NaCl 0.9%, whose half-life was 14.63 min.

**Figure 7:**
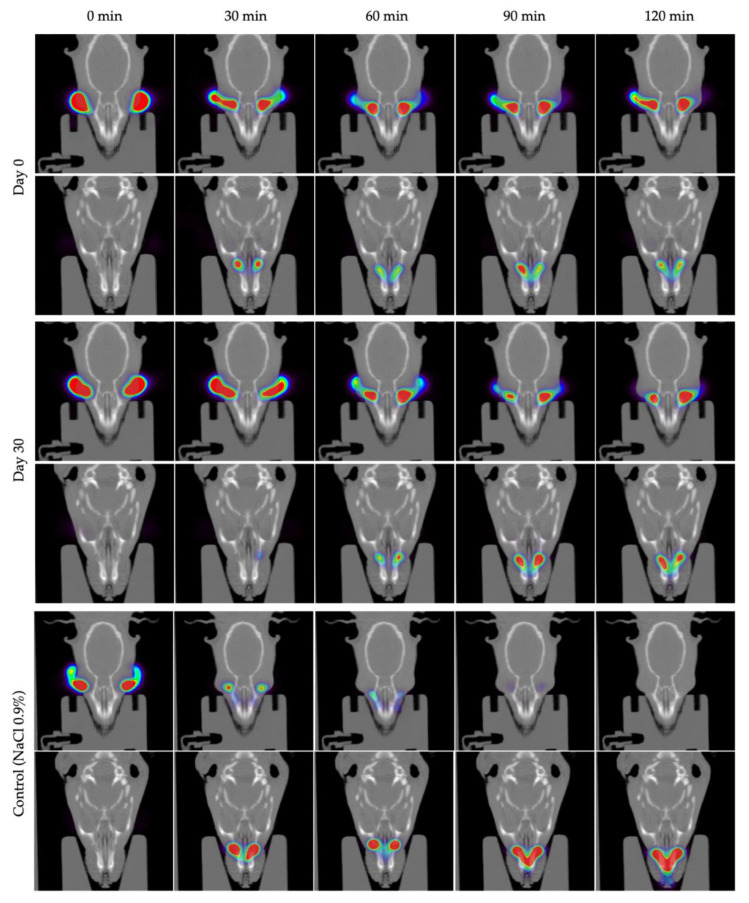
Positron emission tomography (PET) images (maximum intensity projections, coronal views) obtained at different time points after ocular administration of eye drops labelled with ^18^F-fluorodeoxyglucose (^18^F-FDG).

Figure 7 shows an axial view of the PET registration images of animal heads immediately after administration and 30, 60, 90, and 120 min after instillation, showing the distribution of the labelled formulation. Initially, the formulation is located on the surface of the eye. After 2 h, the concentration in the eye was still significant, indicating a long retention time on the ocular surface. An additional radiotracer signal was detected in the nasolacrimal duct and nasal cavity, as part of the formulation entered the nasal cavity from the lacrimal sac. 

## 4. Discussion

Currently, only two commercial ophthalmic presentations of cysteamine (Cystaran^®^ and Cystadrops^®^) are authorized for the treatment of cystinosis, but they are not commercialized in all countries [25]. Thus, pharmaceutical compounding from HPD can constitute a therapeutic alternative, avoiding the difficulty of access to treatment by some patients [46].

Undoubtedly, the physicochemical instability of cysteamine has been one of the greatest challenges when developing ophthalmic formulations with an acceptable period of validity that allows for adequate therapeutic compliance and avoids numerous hospital visits to collect the medication. Cysteamine is very unstable in a solution, where it rapidly converts to cystamine, a non-toxic molecule, but without therapeutic effect. Several strategies have been shown to effectively reduce cysteamine oxidation, such oxygen removal, decreasing temperature, the addition of antioxidant compounds—such as ascorbic acid or disodium edetate (EDTA)—or solution acidification [24,25,47]. To date, several stability studies of cysteamine formulations have been published [6,20,21,25,48], but the lack of uniformity in their preparation (vehicles, excipients, packaging, storage conditions, etc.) and the different determination methods used have led to irreproducible results.

The aim of the present work is to find a simple method of elaboration and conservation of an ophthalmic compounded formulation of cysteamine for an adequate translation to clinical practice. This is intended to offer a practical solution to processing services and to facilitate dispensing of the drug to patients. Our results showed that the new vehicle and single-dose packaging system used in this work allowed the treatment to be dispensed for a long period of time, as long as the containers were stored under low-temperature conditions. With this innovative system, it is possible to use one vial per day with the possibility of closing it, while the rest of the batch can be kept at a low temperature. On the contrary, the use of multidose vials is associated with greater contact with oxygen after opening, since the air contained inside the container is renewed with each opening, favoring the oxidation of cysteamine. A compounded formulation of 0.55% cysteamine, produced with a commercial preparation of 0.4% HA, was stored under refrigeration (2–8 °C) or freezing (−20 °C). To prevent oxidation, saturation with nitrogen was also studied. Physicochemical, microbiological, and mechanical studies were carried out for a period of 30 days, discarding those storage conditions that were inadequate in achieving a long period of validity, until optimal conditions were achieved. Considering all these data, an in-use stability study was carried out.

For this study, a new determination method was developed based on the one previously proposed by Kim et al. [38], which allows for the detection of both cysteamine and cystamine, in a short analysis period of just 6 min. Our results showed that the cysteamine compounded formulation was not stable beyond 7 days when stored at 2–8 °C in single dose vials, which is impractical for patients and HPDs daily work, but when preserved at −20 °C, a stability of at least 30 days was achieved. These findings are consistent with those previously published in the literature reporting that oxidation to cystamine is favored at high temperatures [21,47,49,50,51]. Reda et al. previously showed the effects of storage in the refrigerator and freezer for up to 52 weeks, where it was seen that conservation at −20 °C guaranteed a longer period of validity [21]. Although the results differ slightly from those of the present work, it is important to consider the differences in terms of the composition of the mixture, final packaging, and determination method.

In addition to temperature, the presence of oxygen has also been described as a relevant factor that plays an important role in the chemical stability of cysteamine [48]. Therefore, in order to prevent oxidation, the saturation of the formulation with nitrogen was also studied. However, in the present study, this strategy did not provide less degradation at any of the temperatures or times studied with respect to formulations without nitrogen, as can be seen in Figure 2. This result can possibly be explained due to the material that makes up the single-dose COL containers, PVC, with known oxygen permeability [52]. This permeability may allow for the passage of oxygen through the material, losing the beneficial effect that the initial saturation of the formulations with nitrogen may have had. For this reason, it is important to consider the functionality and composition of the containers in which cysteamine formulations are packaged. In this regard, Cystaran^®^ is supplied in an opaque, white, low-density polyethylene (LDPE) bottle with a LDPE-controlled dropper tip and closed with a polypropylene screw cap. As LPDE is permeable to oxygen, Cystaran^®^ must be stored frozen between −25 °C and −15 °C to remain stable for over one year [21]. After thawing the bottle, it must be stored at 2 °C to 25 °C and discarded after one week [17]. Regarding Cystadrops^®^, it can be stored for six months refrigerated prior to opening, with no need of being frozen. This is possible due to its packaging, which consist of a sealed amber vial with a bromobutyl stopper and an aluminium seal, which have a very low permeability to oxygen [28,49]. Before opening the bottle, the seal and stopper must be removed and replaced by a PVC dropper with a high-density polyethylene (HDPE) closure, which mandates that the bottle must be discarded after one week once opened (storage temperature <25 °C) [19]. Regarding the single-dose PVC system used in the present study, its oxygen permeability does not entail a problem for the stability of the formulation when it is frozen. For this reason, nitrogen saturation is ruled out to increase stability in the monodose vials. Furthermore, nitrogen is a resource that is scarcely available in HPDs, adding significant complexity to the manufacturing process.

Regarding the vehicle used for the elaboration, HA was chosen because it has several advantages. On the one hand, it alleviates part of the irritative symptomatology previously described in other cysteamine formulations [53,54,55,56]. This is achieved due to its restorative properties that enhance corneal epithelium healing, improve the function of the ocular surface, increase the stability of the precorneal tear film, and protect/restore the morphology and distribution of the goblet cells responsible for the secretion of mucins [57,58,59,60]. On the other hand, HA reduces the ocular clearance of cysteamine by increasing its retention time on the ocular surface, favoring its ocular bioavailability, as has been demonstrated in previous works [6,7]. According to Sandri et al. [61], hyaluronic acid, especially that of low molecular weight (<202 kDa), exhibits penetration enhancement properties comparable to, or even better than, chitosan hydrochloride, depending on the compound. In this way, cysteamine permeates into the cornea and reaches the lysosomes of the corneal cells where cystine crystals accumulate [14]. Aquoral^®^ has been selected in this study as a source of HA because it is classified as a medical device used for the treatment of dry eye, which allows for keeping both the origin and the molecular weight of HA constant, something that would be more difficult to achieve if HA was obtained as a raw material due to the high variability reported between different manufacturers and batches [32,57]. According to previous publications, these variations may seriously interfere with compounding and stability studies, as they would no longer be reproducible [32,33,34,35].

Pescina et al. showed that cysteamine oxidation decreases at an acidic pH of 4.2, being a pH-dependent reaction, while corneal penetration was found to be poor at pH 4.2, but enhanced at pH 7 [47]. With this reasoning, it might be hypothesized that better cysteamine stability results could be achieved by lowering the pH of ophthalmic preparations, but it is necessary to consider that ocular administration of solutions with a pH too far from the tear pH (7.4–7.7) could cause discomfort in the eye. Formulated with a pH of 4.1–4.5, Cystaran^®^ used this strategy to reduce cystamine degradation [17,47,62]. This correlation between pH and cysteamine stability agrees with the European Assessment report regarding Cystadrops^®^ (pH 4.6–5.4), which states that low buffered pH, along with high viscosity, are the contributing factors to ocular irritation and inflammation, but this has to be weighed against the benefits of the stability of the active substance [53]. Both presentations use benzalkonium chloride as a preservative and as a trans-corneal permeation enhancer, which can produce toxic effects [40]. The most reported ocular adverse events (incidence around 10% or greater) were eye pain, blurred vision, eye irritation, ocular hyperaemia, eye pruritus, increased lacrimation, eye deposit, and instillation site discomfort [2,22,54,55], many of which are related to the pathology itself. In the case of commercial presentations, pH reduction below physiological values was chosen in order to improve the stability of cysteamine with the consequent problems that this entails. On the contrary, in the present work, a pH value to the physiological value has been chosen, with constant values around 6.7. These values are achieved because of the citric acid/citrate buffer system present in the Aquoral^®^ commercial formulation [45], avoiding the excessive tearing associated with formulations with a lower pH [40]. The present proposal for distribution in single-dose containers allows us to maintain acceptable cysteamine stability at a physiological pH due to freezing effects, which also favors the corneal permeation of the active ingredient [47]. 

Regarding osmolality, it remained practically constant throughout the study in all formulations, with values around 350 mOsm/Kg, similar to those previously published [50]. Although these values are higher than the 289 ± 21 mOsm/L reported for tears, considerably higher values are found in the commercial formulations of other active ingredients used in the treatment of chronic ocular pathologies, such as Travatan^®^ (travoprost) or Vividrin iso EDO^®^ (chromoglicin acid), with 368.5 and 341 mOsm/L, respectively [41,63].

Although freezing provides chemical stability to cysteamine, preventing its rapid degradation, it is unknown if this process can affect the physical properties of the formulation itself or of the final packaging. To determine whether this process significantly affected the properties of HA, viscosity studies were carried out on the day of its preparation, as well as after 15 and 30 days of storage in the refrigerator/freezer. From these data, it was possible to see that the viscosity remained practically constant at 2–8 °C, while on days 15 and 30, a drop was observed in those stored at −20 °C. This may be explained due to changes in polymer conformation during the freeze/thaw processes [64]. The present cysteamine compounded formulation maintained the pseudoplastic behavior of HA previously described in the literature [65,66,67] during all storage times and conditions, by decreasing its viscosity with increasing shear force, as seen in Figures S3–S5. 

The clinical implications that the drop in viscosity of the frozen formulations may have on ocular biopermanence is unknown, so we decided to carry out a study using an in vivo molecular imaging technique with the most promising formulation in terms of stability. For determination, 0.9% NaCl was used as a control, as it is one of the most widely used vehicles in the preparation of eye drops compounded formulations produced in HPD [40,68]. Our results showed that the decrease in the viscosity of the frozen formulations did not translate into relevant clinical changes, and their bioadhesive properties were maintained. This was demonstrated by the lack of relevant differences in ocular biopermanence between the formulations made with AQ before and after thawing. These ocular biopermanence results are in line with those previously published [6]. Another relevant aspect that we wanted to assess with this work was whether the single-dose container could be altered by the freezing/thawing process. We observed that the elasticity of the single-dose vials remained constant after 30 days of storage, both in the refrigerator and in the freezer, despite the increase in the internal volume caused by the freezing of the content inside the containers.

After characterizing the formulation under different storage conditions for 30 days, its chemical stability in-use after opening was determined. Conditions were established to simulate its use in real life, opening the single-dose container 5 times a day and storing it at room temperature. We have confirmed that the vial can be used within the first 16 h of the day after 30 days of storage in the freezer. This allows for convenient transport, thanks to its portability, with no need for refrigeration, thus facilitating the administration of the drops throughout the day, therefore increasing the therapeutic adherence of the patients. These results cannot be compared with those previously published by Martín-Sabroso et al. due to methodological differences. The previous work used a different vehicle and final containers and performed the stability study using vials stored in the refrigerator and opened 4 times a day for 7 days, where degradation ranging from 30% to 60% was observed for all the formulations after 7 days of use [48].

Regarding the limitations of the present study, some aspects of the formulation and the vials used have not been characterized in depth. For instance, the behavior of HA after the freeze/thaw process requires further studies to better characterize the vehicle. A cryoprotective compound could be used as an excipient in future formulations to prevent the properties from being modified by freezing [69]. Moreover, measuring the oxygen permeability of the single-dose container would explain why bubbling with nitrogen provides scarcely any improvement in the chemical stability of cysteamine. Finally, it would also be interesting to know whether this parameter is affected by the freezing/thawing processes.

## 5. Conclusions

Hospital pharmacy departments play a relevant role in facilitating access to drugs when these are not readily available in all countries, such as orphan drugs. Cysteamine eye drops are used to treat ocular cystinosis, which is an easily oxidizable molecule that requires rigorous stability studies to establish an adequate period of validity. With the aim to find a simple method of preparation and conservation of an ophthalmic compounded formulation of 0.55% cysteamine in HA, packaging in single-dose containers and subsequent freezing for 30 days, protected from light, are proposed. These conditions preserved the stability and biopermanence of the formulation and the integrity of the packaging material, which was scarcely affected by the low temperatures. Once thawed, vials can be used for 16 h, allowing for the administration of cysteamine every 4 h. These results favor a quick and easy transfer to clinical practice, eluding complex production processes such as nitrogen saturation, and avoiding numerous hospital visits to collect the compounded formulation, facilitating better therapeutic adherence of patients to treatment. 

## Figures and Tables

**Table 1:** Gradient UHPLC quantification method for cysteamine and cystamine. The mobile phase A was an aqueous solution of 4 mM sodium heptane sulfonate, and the mobile phase B was acetonitrile.

Time	Mobile Phase A (%)	Mobile Phase B (%)
0	95.0	5.0
0.50	95.0	5.0
2.50	70.0	30.0
3.20	95.0	5.0
6.0	95.0	5.0

**Table 2:** Degradation rate constants (*K*) from storage stability studies. *SDk*: standard deviation of *K*; *r*^2^: goodness-of-fit measure for linear regression models.

Storage Conditions	*K* (%/Day)	*SDk* (%/Day)	*r* ^2^
2–8 °C	1.414	0.05502	0.9270
2–8 °C nitrogen	1.496	0.02904	0.9808

## Data Availability

Not applicable.

## Supplementary Materials

The following are available online at https://www.mdpi.com/article/10.3390/pharmaceutics14102194/s1, Figure S1: COL Eye Drops System^®^ used in the stability study, provided from Biomed Device; Figure S2. Cystamine content with respect to the initial concentration throughout the study for each of the temperature settings (refrigeration/freezing) and presence/absence of nitrogen; Figure S3. Speed-scan test of the cysteamine formulations at 25 °C with the presence/absence of nitrogen at day 0, where the viscosity is measured at different revolutions per minute (rpm); Figure S4. Speed-scan test at 25 °C of the cysteamine formulations stored at different temperature conditions and in the presence/absence of nitrogen at day 15, where the viscosity is measured at different rpm; Figure S5. Speed-scan test at 25 °C of the cysteamine formulations stored at different temperature conditions and in the presence/absence of nitrogen at day 30, where the viscosity is measured at different rpm.
