# Supplementary material for: Cysteamine Eye Drops in Hyaluronic Acid Packaged in Innovative Single-Dose Systems: Stability and Ocular Biopermanence
Source: Pharmaceutics. 2022 Oct 15;14(10):2194. doi: 10.3390/pharmaceutics14102194 (PMC9607622; doi:10.3390/pharmaceutics14102194)
Supplement: Supplementary file 1 [file pharmaceutics-14-02194-s001.zip › pharmaceutics-1959226-supplementary.pdf]

pharmaceutics MDPI

Supplementary Material: Cysteamine Eye Drops in Hyaluronic Acid Packaged in Innovative Single-Dose Systems: Stability and Ocular Biopermanence

Ana Castro-Balado [1,2,3†], Enrique Bandín-Vilar [1,2,3†], Andrea Cuartero-Martínez [2†], Laura García-Quintanilla [1,2,3]; Gonzalo Hermelo-Vidal [2], Xurxo García-Otero [3,4], Lorena Rodríguez-Martínez [2], Jesús Mateos [2], Manuela Hernández-Blanco [5], Pablo Aguiar [4], Irene Zarra-Ferro [1,2], Miguel González-Barcia [1,2], Cristina Mondelo-García [1,2,*], Francisco J. Otero-Espinar [3,*] and Anxo Fernández-Ferreiro [1,2,*]

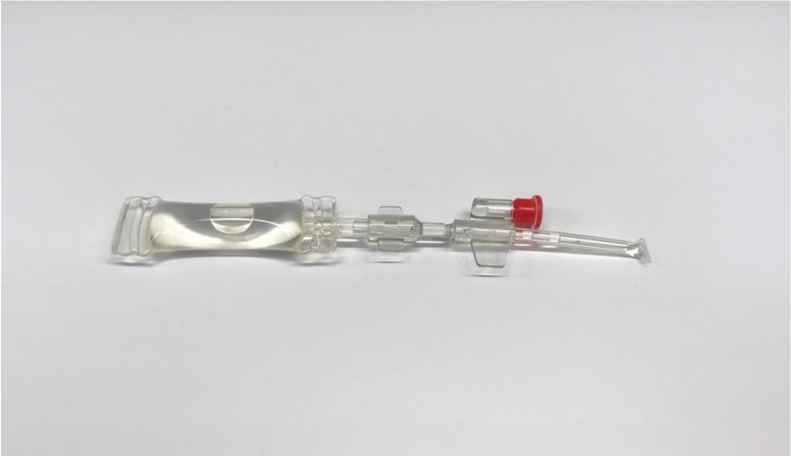

Figure S1. COL Eye Drops System® used in the stability study, provided from Biomed Device.

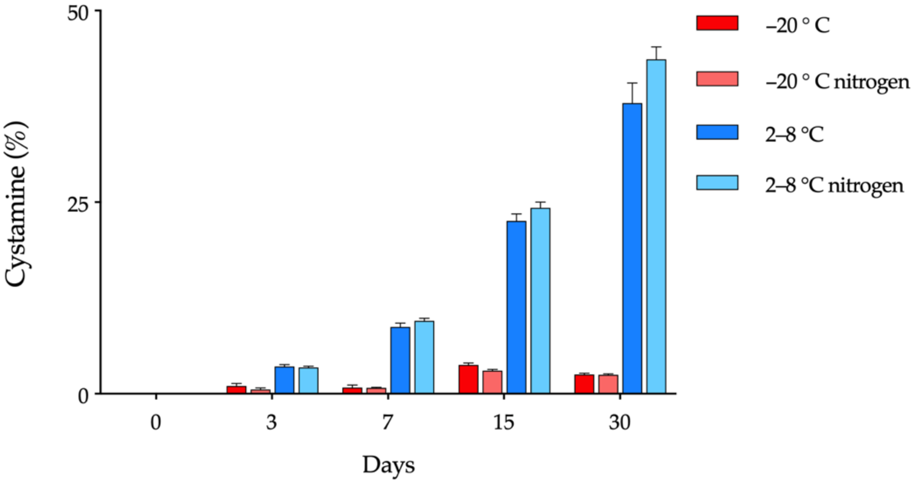

Figure S2. Cystamine content with respect to the initial concentration throughout the study for each of the temperature settings (refrigeration/freezing) and presence/absence of nitrogen.

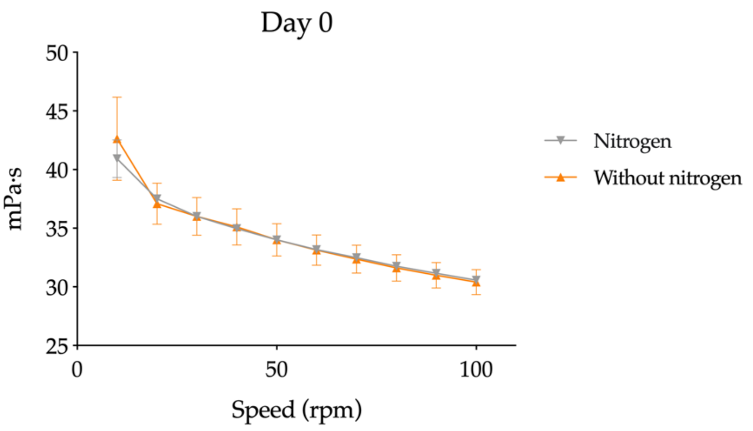

Figure S3. Speed-scan test at 25 °C of the cysteamine formulations with presence/absence of nitrogen at day 0, where the viscosity is measured at different revolutions per minute (rpm).

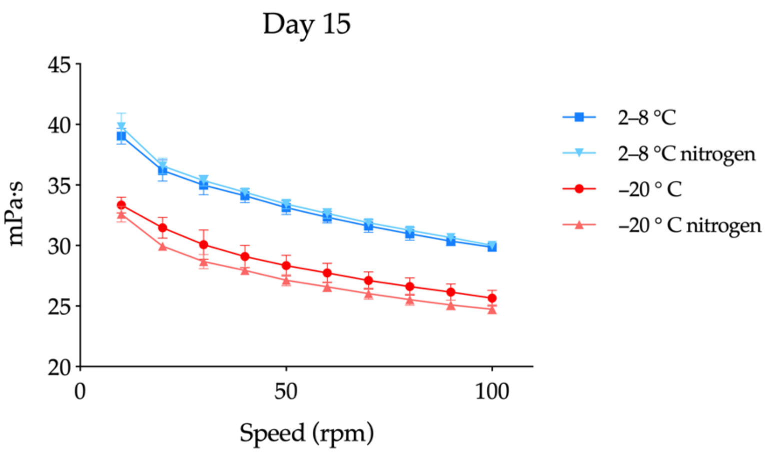

Figure S4. Speed-scan test at 25 °C of the cysteamine formulations stored at different temperature conditions and presence/absence of nitrogen at day 15, where the viscosity is measured at different rpm.

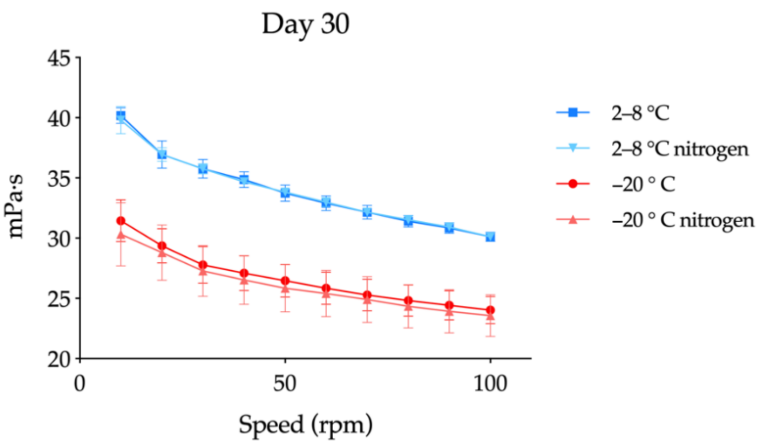

Figure S5. Speed-scan test at 25 °C of the cysteamine formulations stored at different temperature conditions and presence/absence of nitrogen at day 30, where the viscosity is measured at different rpm.
